# Targeted Swabbing of Implant-Associated Biofilm Formation—A Staining-Guided Sampling Approach for Optimizing Routine Microbiological Diagnostics

**DOI:** 10.3390/diagnostics11061038

**Published:** 2021-06-04

**Authors:** Sylvio Redanz, Andreas Enz, Andreas Podbielski, Philipp Warnke

**Affiliations:** 1 Institute of Medical Microbiology, Virology, and Hygiene, University Medicine Rostock, Schillingallee 70, 18057 Rostock, Germany; sylvio.redanz@gmx.de (S.R.); andreas.podbielski@med.uni-rostock.de (A.P.); philipp.warnke@med.uni-rostock.de (P.W.); 2 Department of Translational Rheumatology and Immunology, Institute for Musculoskeletal Medicine, Medical Faculty Münster, Albert-Schweitzer-Campus 1, 48149 Münster, Germany; 3 Orthopaedic Clinic and Policlinic, University Medicine Rostock, Doberaner Str. 142, 18057 Rostock, Germany

**Keywords:** implant, swab, diagnostics, biofilm, staining, PJI

## Abstract

**Background:** Swabbing of implants removed from potentially infected sites represents a time saving and ubiquitously applicable alternative to sonication approaches. The latter bears an elevated risk of processing related contaminations due to the high number of handling steps. Since biofilms are usually invisible to the naked eye, adequate swabbing relies on the chance of hitting the colonized area on the implant. A targeted directed swabbing approach could overcome this detriment. **Method:** Three dyes were tested at different concentrations for their toxicity on biofilm-associated cells of *S. epidermidis*, the species most frequently identified as a causative agent of implant-associated infections. **Results:** Malachite green (0.2%) delivered the highest bacterial recovery rates combined with the best results in biofilm visualization. Its suitability for diagnostic approaches was demonstrated for smooth and rough implant surfaces. Biofilm-covered areas were successfully visualized. **Conclusion:** Subsequent targeted swab-sampling resulted in a significantly increased bacterial recovery rate compared to a dye-free “random swabbing” diagnostic approach.

## 1. Introduction

Implant-associated infections are a worldwide problem often traced back to biofilm formation started by single, surface colonizing and thereafter biofilm-forming bacteria. Since such biofilm-organized bacteria are extremely resistant to host defence mechanisms as well as to antibiotic treatment [1], it is crucial to prevent this initial colonization step and to allow for a fast incorporation of the prosthesis into the host tissue. The underlying process was described in the late 80s as “the race for the surface” [2]. One approach for protecting implants from biofilms is the manipulation of the implant surface. For this purpose, surfaces were altered, for example, by coatings releasing antibacterial substances such as silver/copper ions or by changing the surface structure [3,4,5], thereby enabling a faster integration into the host tissue. Despite all efforts, biofilm formation on implants and subsequent periprosthetic infections, until now, could not be completely prevented. Therefore, explantation and replacement of the infected prostheses are often the last resort. 

To prevent a direct re-colonization of the new implant, the optimum choice of an antibiotic therapy before and after the implant replacement is important. For a specific therapy, the latter decision directly depends on an optimized microbiological diagnostic.

Diagnostics of implant-associated infections usually rely on the combination of periprosthetic tissue sampling and investigations of the implant itself [6,7,8]. Since resident microorganisms being organized in biofilms are the causative agent of these infections, investigation of the implant material itself is most promising. According to the current literature, implant sonication is the state-of-the-art method but requires special technical equipment and extended periods of hands-on time [9,10,11,12,13,14]. The inherent high number of working steps also bear the risk of introducing contaminations. For these reasons, not all diagnostic laboratories provide this technique. Alternatively, traditional swab-based sampling of the implant material could be a suitable method, provided the appropriate swab type is chosen. It can be performed by every lab and is cost efficient due to the simple equipment and the short and easy processing compared to sonication procedures. 

Bacterial biofilms are usually not visible to the naked eye. In addition, they do not necessarily colonize the entire implant but instead are located only on specific areas of the implant. As a consequence, especially biofilm growth limited to such areas could remain undetected by a random swabbing approach. Biofilm-staining could increase the chances to identify statistically dispersed biofilm areas. Therefore, such spots could be sampled in a targeted mode and thus could contribute to an increased microbial recovery rate, which in turn is the most important sensitivity determinant in culture-based diagnostics. With the intention to improve both the reliability of swab-based implant material sampling and the survival of stained bacteria, three different dyes in different concentrations were tested. Safranin O and crystal violet are used for Gram staining and are therefore present in every diagnostic laboratory. Their suitability for biofilm staining was previously demonstrated for various microorganisms [15,16,17,18,19]. In addition, malachite green was selected, another dye which is commonly used in dentistry due to its high contrast visualization of oral biofilms [20,21]. However, it is also known to be toxic in higher concentrations [22]. 

Therefore, this study had two major aims: first, to elucidate the bacterial recovery after staining with the chosen chemicals, and second, to demonstrate its routine diagnostic suitability with commonly used implant materials employing a previously established in vitro model for implant associated infections [23]. 

## 2. Materials and Methods

### 2.1. Bacterial Growth Conditions

*S. epidermidis* RP62A (ATCC 35984; American Type Culture Collection, Manassas, USA) was routinely grown in tryptic soya broth medium (TSB, CASO-bouillon, ROTH GmbH, Karlsruhe, Germany). Static cultures were incubated at 37 °C and under a 20% O_2_/5% CO_2_ atmosphere. Columbia agar plates (BD, Heidelberg, Germany) were used for CFU determination and incubated for 24 h prior to counting. Biofilms were grown in accordance with a previously published in vitro test model for implant associated infections [23].

### 2.2. Biofilm Growth on Titanium Alloy Specimens 

For staining experiments, biofilms were grown on titanium alloy, as described before [23]. Briefly, early stationary phase cells were harvested, washed in sterile phosphate-buffered saline (PBS; NaCl (137 mmol/L), KCl (2.7 mmol/L), Na_2_HPO_4_ × 2 H_2_O (10 mmol/L), KH_2_PO_4_ (2.0 mmol/L); pH 7.4), and adjusted to an optical density (OD600) of 0.5 to reach a cell density of approximately 1 × 10^5^ CFU. Fresh medium was inoculated with 10% (*v/v*) of the OD-adjusted cell suspension. Titanium alloy specimens (TiAl6V4, DOT GmbH, Rostock, Germany; 11 mm diameter × 2 mm) with a roughness of approximately Rz = 4 μm were placed in 24-well polystyrene plates, overlaid with 1 mL of inoculated medium and incubated at 37 °C under a 20% O_2_/5% CO_2_ atmosphere for 3 days. 

### 2.3. Biofilm Staining on Titanium Alloy Specimens 

Biofilm carriers were washed intensively in PBS to eliminate planktonic bacteria. Therefore, the carrier was grabbed with a sterile forceps and repeatedly submerged in sterile PBS (20 times) for 10 s. This washing procedure was performed twice. Washed titanium alloy discs were allowed to dry at room temperature and ambient air for 30 min. Biofilms were stained with safranin O, crystal violet, or malachite green. Therefore, 10 µL of safranin O (0.1 and 1.0% *w/v*), crystal violet (0.1 and 1.0% *w/v*), and malachite green (0.2 and 2.0% *w/v*) were applied to the biofilm. After 3 min, dyes were removed by repeatedly washing in PBS as described above and allowed to dry at room temperature and ambient air. As a reference, additional discs were treated accordingly in bacteria-free medium. 

### 2.4. CFU Determination after Biofilm Staining on Titanium Alloy Specimens 

**Figure 1:**
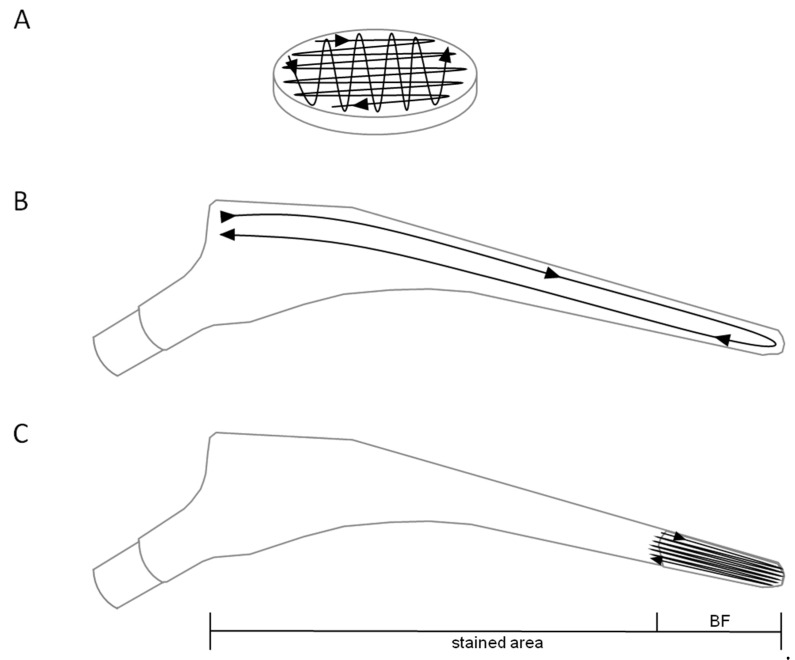
Schematic display of the swabbing procedure. Shown are swabbing paths for biofilm sampling from titanium alloy biofilm carriers (**A**) and hip implant material, before (**B**) and after (**C**) biofilm visualization. Staining area and biofilm (BF) area are indicated below.

Biofilms were swabbed with Sigma Swab (cellular foam, MWE medical wire, Corsham Wiltshire England; ref. MW941) as follows: Biofilm carriers were locked into position with a sterile forceps. The swab was repeatedly moved over the full width of the biofilm carrier for 10 s (20 streaks, Figure 1A), a constant contact pressure of 200 mN (±20 mN), and continuously rotating the swab. Subsequently, CFUs were determined by the roll-plate method (as recommended by the CLSI [24]) and subsequent counting. Therefore, swabs were rolled in 30 dense streaks over Columbia agar (20 exposing the side while constantly rotating the swab and 10 exposing the tip to the agar surface). To ensure the same conditions for every single swabbing process a single previously trained person performed all experiments under standardized conditions. Constant contact pressure was confirmed by performing sampling on a scale.

### 2.5. Biofilm Growth and Staining on Hip Implant Material 

Two different implant surfaces were tested for the applicability of biofilm staining for diagnostic approaches: (i) LCU hip prosthesis (Waldemar Link GmbH, Hamburg, Germany), which is characterized by an increased surface roughness and is intended to be used for cement-free implantation (TiAl6V4 forged alloy, surface processing: corundum-blasting and calcium phosphate coating); (ii) standard C Cem hip prosthesis (Waldemar Link GmbH, Hamburg, Germany), which has a high polished surface (steel alloy FeCrNiMnMoNbN), designed to be implanted in the proximal femur with polymethylmethacrylat (PMMA) bone cement. 

For biofilm growth, an overnight culture of *S. epidermidis* RP62A was diluted 1:40 in fresh medium. Implants were placed into flasks so that inoculated medium covered 2 cm of the distal section. After incubation for 3 days, implants were removed from flasks and extensively washed in PBS and dried as described above. Before staining, surfaces were swabbed twice over the full length of the implant (12 cm, Figure 1B) by continuously rotating the swab. Two sides per implant were swabbed separately (one swab per wide and slim side). 

Subsequently, biofilms were stained by submerging the implant for 20 s in 0.2% (*w/v*) malachite green followed by washing and drying as described above. Targeted swabbing of stained biofilms was performed similarly to sampling from unstained biofilms. However, instead of sampling the whole implant surface, swabbing was restricted to the area of visible biofilm growth. Since the biofilm area spanned 2 cm of the whole implant, swabbing was done repeatedly to cover the same distance as without staining (Figure 1C). 

Swabbing after staining was performed on the opposite side of the implant compared to the pre-staining procedure. Thus, post-staining swabbing was not biased by a reduced bacterial load resulting from pre-staining swabbing. Swabs were then homogenized and microbiologically processed by a semi-automated homogenization method as described elsewhere [25]. Serial dilutions were plated on Columbia agar in replicates. CFUs were counted the next day. Replicates were averaged. Each data point represents the bacterial load of one swab. 

### 2.6. Statistics and Reproducibility 

Data of at least 4 independent experiments were analyzed using SPSS. Statistical differences were determined by Student’s *t*-test and *p*-values < 0.05 were considered as significant. 

### 2.7. Regeneration of Titanium Alloy Specimens and Implant Materials 

After swabbing, all biofilm carriers were placed in 70% (*v/v*) ethanol for at least 24 h. Subsequently, biofilm residues were removed by sonication for 30 min at 100% (35 kHz, 320 W, Sonorex Digital P10, Bandelin Electronic GmbH & Co. KG, Berlin, Germany) following the manufacturer’s instructions. Discs and implants were subsequently flushed with deionized water and sterilized by autoclaving.

## 3. Results

### 3.1. Visualization of Biofilms on Titanium Alloy Disc

Standard concentrations of safranin O and crystal violet for biofilm staining are 0.1% (*w/v*) [18]. To increase contrast, 10-fold elevated concentrations were tested as well. Malachite green is recommended to be used as a 2% (*w/v*) [20] solution for optimal biofilm staining. Considering its toxicity, a 10-fold lower concentration was tested for its staining capacity. 

**Figure 2:**
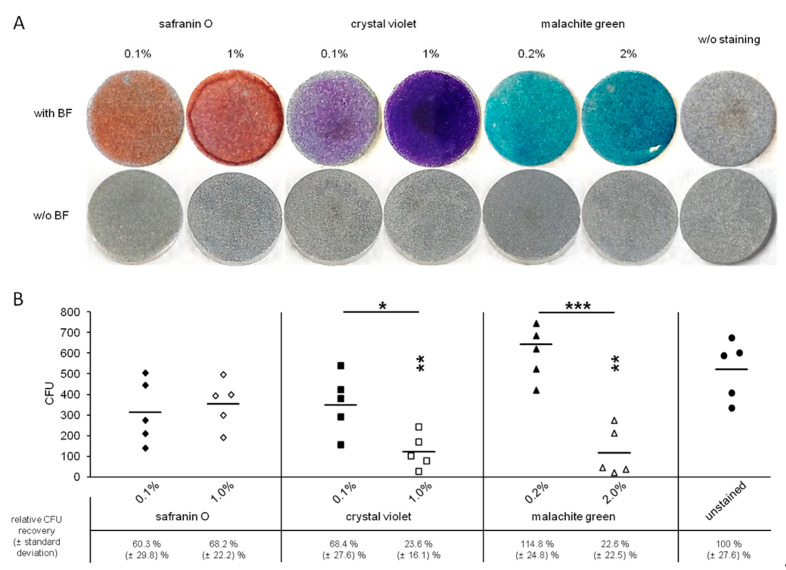
Staining and sampling of titanium alloy biofilm carriers. *S. epidermidis* biofilms (BF) were grown for three days on titanium alloy specimens. Biofilms were stained with safranin O (0.1% and 1% (*w/v*)), crystal violet (0.1% and 1% (*w/v*)), or malachite green (0.2% and 2% (*w/v*)). Representative images of stained biofilms (upper row) as well as of stained biofilm-negative titanium alloy specimens (lower row) are given (**A**). Biofilms were sampled by swabbing and subsequently microbiologically analyzed by roll-plate method on Columbia agar plates. Recovered CFUs and geometric means are given in the lower panel (**B**). Each staining setup was compared to unstained control discs. The respective significance levels are indicated by asterisks above each column. Significance levels between different stain concentrations are given above each pair of columns and indicated by a bar. Significance levels are as follows: * *p* < 0.05; ** *p* < 0.01; *** *p* < 0.001 (Student’s *t*-test, unpaired, two-tailed, heteroscedastic). Relative CFU recovery rates compared to unstained control discs are given below the graph. Shown are data of five independent experiments.

All concentrations of the tested staining solutions were suitable to visualize *S. epidermidis* biofilm formation (Figure 2A). The absence of staining artefacts was evaluated by testing biofilm-free discs in the same manner as biofilm bearing discs; none of the tested staining solution resulted in unspecific coloration of the biofilm carrier itself. 

### 3.2. Toxicity of the Staining Solutions 

Staining and subsequent CFU determination of biofilms revealed that crystal violet as well as malachite green is toxic to biofilm bacteria in higher concentrations (1 and 2%, respectively) as recovered CFUs were significantly lower compared to the no staining setup (Figure 2B). The reduction of the stains’ concentrations resulted in a significant increase of the number of recovered bacteria. Interestingly, although not significantly different compared to unstained control discs, CFUs levels were still lower in the safranin O (0.1% and 1.0%) and 0.1% crystal violet setup. Only 0.2% malachite green staining led to a comparable CFU recovery rate as the unstained controls.

### 3.3. Visualization of Biofilms on Implant Materials 

**Figure 3:**
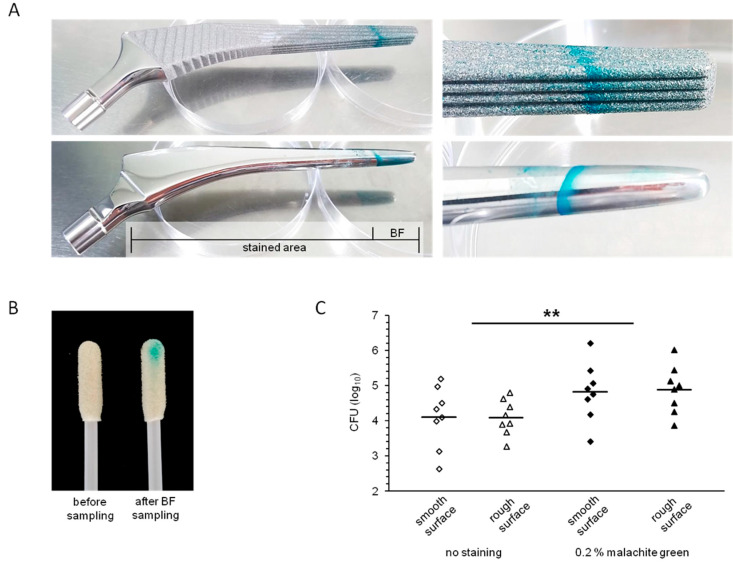
Staining and sampling of hip implant material. Biofilms (BF) of *S. epidermidis* were grown on two different hip implant materials: (i) LCU hip prosthesis (TiAl6V4 forged alloy, increased surface roughness) and (ii) standard C Cem hip prosthesis (polished steel alloy FeCrNiMnMoNbN). Biofilms were grown for three days and subsequently stained with malachite green (0.2% (*w/v*)). Representative images of 4 independent experiments are given in the upper panel. Stained area and area of biofilm growth are indicated. A magnified close-up is shown on the right (**A**). Representative swabs before and after biofilm sampling are displayed (**B**). Recovered CFUs are given in log10 scale in the lower panel (**C**). Every experiment included two samplings per condition resulting in 8 data points each. Geometric means are indicated. Significance levels were calculated comparing stained biofilms with the non-staining setup. ** *p* < 0.01 (Student’s *t*-test, unpaired, two-tailed, heteroscedastic).

Staining with 0.2% malachite green resulted in adequate visualization with a high contrast resolution of *S. epidermidis* biofilms (Figure 3A). In addition, due to sample uptake, the swab became colorized (Figure 3B), which further ensures adequate sample uptake. 

### 3.4. Recovery of Biofilm Bacteria from Implant Surfaces 

Stain-directed sampling of biofilm cells resulted in an elevated CFU count (Figure 3C). On average, cell numbers (determined as CFUs) recovered from smooth or rough surfaces were approximately 5- or 6-times higher, respectively, after staining compared to the non-stained setup. Analyzing the results from both surfaces, the differences were statistically significant (*p* = 0.006).

## 4. Discussion

Adequate sampling is essential for the outcome of every diagnostic test. Often, sampling or sample preparation is suboptimal and results in a low recovery rate of microorganisms, especially with respect to swab-based sampling [26,27]. For detecting implant-associated infections, several methods for collecting microorganisms were tested. These included intra-surgical swabbing, sonication of implant materials or the collection of periprosthetic tissue for cultural and histological examinations [6,7,8,14]. All these approaches have their pros and cons; while sonication bears a considerable risk of contamination due to the high number of handling steps [12], the other methods rely on sampling of the correct area. This is a major pitfall, since not all periprosthetic infections affect the whole implant surface. 

Implant-associated infections are often caused by microorganisms organized in biofilms. These biofilms are extremely resistant to host defence mechanisms as well as to antibiotic treatment. For therapeutic success, biofilm-covered implant materials are thus frequently treated by removing the entire prosthesis [28]. Yet, biofilm growth of the causative agents is also a chance for special diagnostic approaches. Therefore, sampling from the implant surface bears a diagnostic advantage over other approaches. 

A cost-efficient and less time-consuming alternative to implant sonication is a swab-based approach which could be performed by every diagnostic laboratory. However, currently, choosing the sampling area is done more or less randomly in most cases since the biofilm is usually not visible to the naked eye. 

Different surfaces as well as diverse environments do have specific effects on biofilm formation. These circumstances cannot be comprehensively addressed in a laboratory setting. Therefore, we focused on the most common types of implant surfaces, i.e., smooth and rough. Here we describe a simple, reliable and cost-efficient method for biofilm visualization, which offers the possibility of directed and precise sampling. The location of the biofilm can easily be determined, which results in accurate sampling of the true area of biofilm growth instead of randomly sampling the whole implant. In addition, since uptake of the biofilm sample resulted in the colorization of the used swab, this approach inherently helps to control successful sampling. This also improves downstream microbiological processing: The roll-plate method could be performed in a more directed way, since the potential bacteria coated side of the swab is visible. 

Staining of the biofilm enabled directed sampling and, thus, resulted in significantly increased bacterial recovery rates. This is a major benefit of this approach, since most often only low amounts of viable bacteria are present due to an altered bacterial metabolism (viable but non-culturable (VBNC) bacteria in biofilms) as well as an antibiotic pre-treatment [29,30,31]. In addition, the direct visualization of a biofilm covering the implant material inherently contributes to the diagnosis of periprosthetic infection, since culture-based or molecular detection of microorganisms always bears the risk of identifying contaminants introduced during the diagnostic procedures. 

The concept of the current study was proof-of-principle for biofilm visualization on typical implant materials and its effect on swab-based sampling and consecutive bacterial recovery employing *S. epidermidis* as a model organism, since it is the bacterial species most frequently recovered from infected implant sites [32,33]. Visualizing biofilms in combination with precise, targeted swabbing is a new and promising approach in routine microbiological diagnostics of implants, but it is still a preliminary method that has to be verified in future investigations including patient-derived material.

## Data Availability

The data were collected and evaluated within the Institute of Medical Microbiology, Virology, and Hygiene, University Medicine Rostock, Schillingallee 70, 18057 Rostock, Germany. The collected data obtained have been stored and are available at the Institute of Medical Microbiology, Virology, and Hygiene.
